# Disseminated neurocysticercosis with bilateral papilledema: a case report

**DOI:** 10.1186/s13256-019-2227-0

**Published:** 2019-09-18

**Authors:** Ruchi Shrestha, Amin Kumar Shrestha

**Affiliations:** 1 Department of Ophthalmology, Reiyukai Eiko Masunaga Eye Hospital, Banepa-1, Kavrepalanchok District, Nepal; 2 Department of Orthopedics, Nepal Police Hospital, Panipokhari, Kathmandu, Nepal

**Keywords:** Ocular cysticercosis, Disseminated subcutaneous nodules, Papilledema

## Abstract

**Background:**

Ocular cysticercosis is a disease which rarely involves cutaneous skin and the optic nerve. Patients with clinical presentation of subcutaneous nodules and papilledema should always be evaluated for cysticercosis.

**Case presentation:**

We report a rare case of ocular cysticercosis with multiple disseminated subcutaneous nodules and papilledema in both eyes. A 22-year-old Brahmin man presented with complaints of gradual loss of vision in both eyes and multiple small masses all over his body. On clinical evaluation, multiple subcutaneous nodules were seen on his face, mandibular area, elbow, arm, and abdomen. A fundus evaluation showed bilateral blurred disc margin. The case was managed with steroids and anti-parasitic drugs.

**Conclusion:**

This case report highlights the importance of ruling out neurocysticercosis in cases with multiple disseminated subcutaneous nodules and papilledema.

## Background

Human cysticercosis is an accidental event, which results from either ingestion of cysticercus larvae in raw or inadequately cooked pork or ingestion of *Taenia solium* in contaminated water, food, and vegetables, or autoinfection due to poor hygiene [1]. Ocular cysticercosis may affect almost all eye tissues. The vitreous cavity, subretinal space, and subconjunctival space are common sites while involvement of other regions (such as extraocular muscle and optic nerve) is relatively less common [2].

We report a rare case of ocular cysticercosis with multiple disseminated subcutaneous nodules on the body with bilateral papilledema with multiple calcified cysts with scolex in brain parenchyma and cerebellum on computed tomography (CT) scan. Ocular cysticercosis is a disease which rarely involves cutaneous skin and optic nerve. This case report highlights the importance of always evaluating patients with clinical presentation of subcutaneous nodules and papilledema for cysticercosis.

## Case presentation

**Fig. 1:**
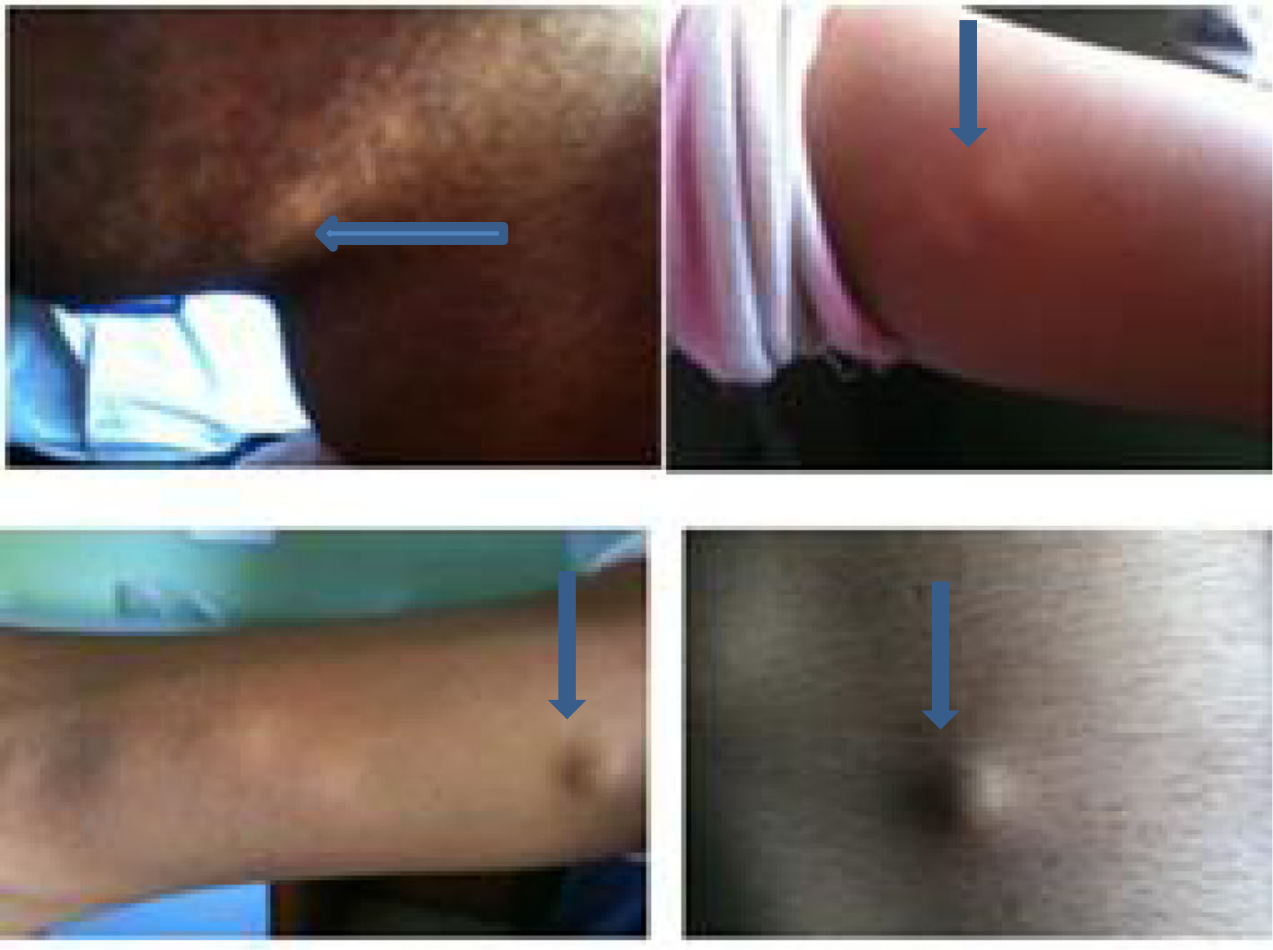
Disseminated subcutaneous nodules as indicated by *arrows* on submandibular area, elbow, arm, and abdomen

A 22-year-old Brahmin man presented with complaints of gradual, painless, progressive loss of vision in both eyes for 1 month. It was associated with complaints of headache and dizziness for 1 month. He had a history of multiple painless swellings over his body for the past 1 year. Multiple swellings were present on his face, mandibular area, elbow, arm, and abdomen. His headache was intense on awakening and bending down position. He had a history of fever for 2 days after intake of anti-filariasis medication 1 month back. There was no history of vomiting, unconsciousness, seizures, or change in behavior. He had a history of using tablet amitriptyline 75 mg before sleep for headache for the past 15 days. He was a plumber by occupation. He did not smoke tobacco or drink alcohol; he was not a vegetarian by diet and had a history of eating wild pork meat. There was no significant history of similar illness in his family. There was no significant social and environmental history. On examination he was healthy and well oriented to time, place, and person. His visual acuity was 6/12 in both eyes. Intraocular pressure was 12 mmHg in both eyes. He had multiple small pea-sized nodules which were soft, mobile, non-tender, and well defined; each nodule was approximately 2 × 2 cm. There were no signs of inflammation on his face, mandibular area, elbow, arm, and abdomen (Fig. 1).

**Fig. 2:**
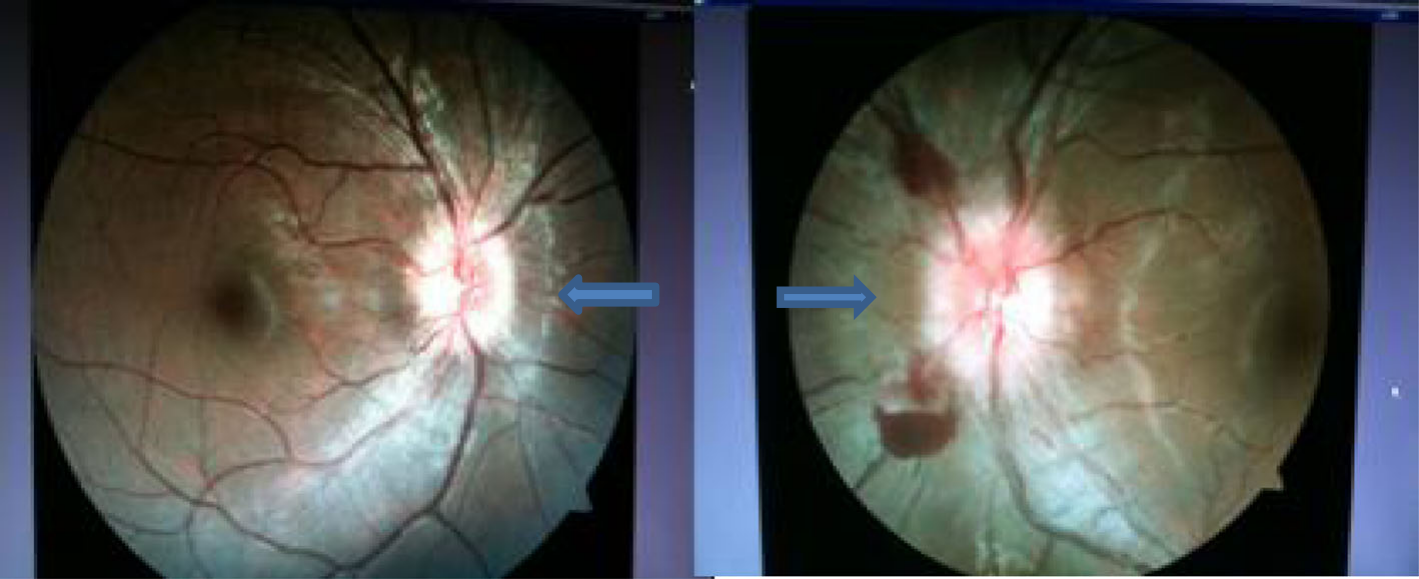
Fundus photograph showing bilateral blurred, elevated disc margin and tortuous dilated vessels with parapapillary hemorrhage in left eye as indicated by *arrows*

His vital signs and systemic examination were normal. He had no neurological deficit. Pupillary reaction was sluggish in both eyes, otherwise the anterior segment was unremarkable. Posterior segment revealed papilledema in both eyes in the form of blurred, elevated disc margin and tortuous dilated vessels. Parapapillary hemorrhage was present in his left eye. The macula was healthy with good foveal reflex (Fig. 2).

**Fig. 3:**
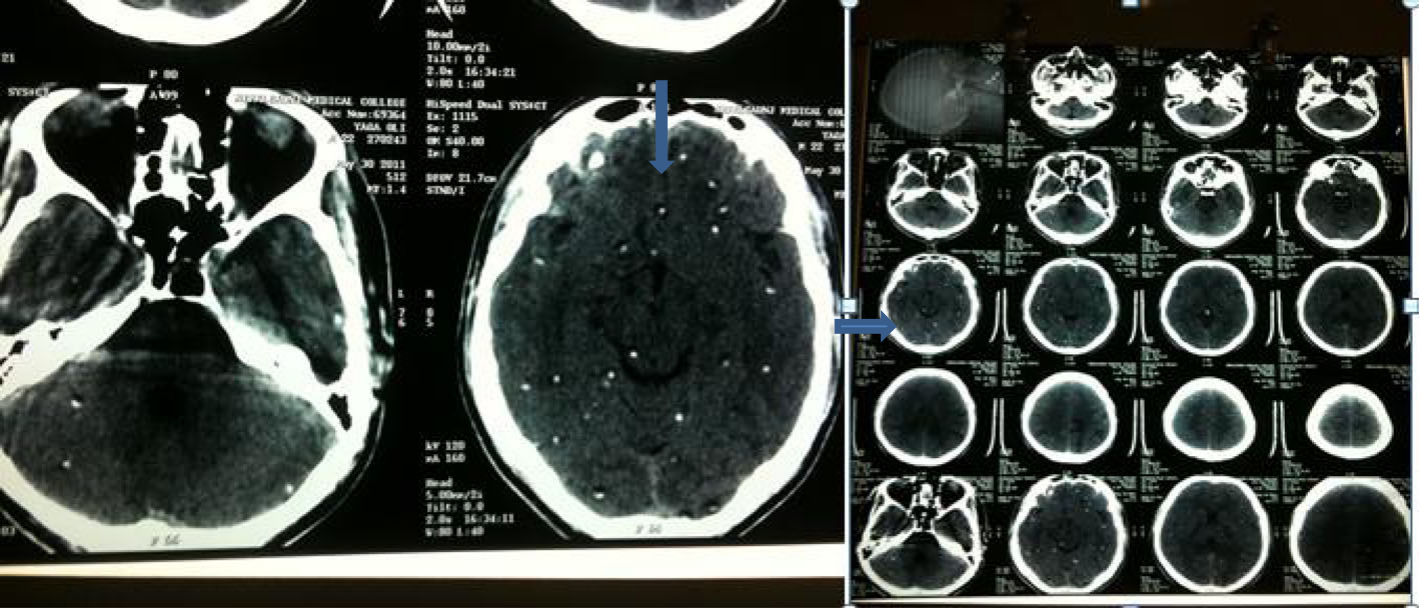
Computed tomography scan of the brain showing multiple calcified lesions with scolex as indicated by *arrows* in cerebellum and brain parenchyma, with multiple sites of edema on left side of parietal area

Blood reports were hemoglobin 11.5 gm%, neutrophils (N) 55%, lymphocytes (L) 22%, eosinophils (E) 12%, monocytes (M) 1%, erythrocyte sedimentation rate (ESR) 30 mm/first hour, total leukocyte count (TLC) 7800 cells/mm^3^, and random blood sugar (RBS) 80 mg%. His serology was negative. His urine routine microscopy was normal. Stool routine microscopy showed absence of parasites. A CT scan showed multiple calcified lesions with scolex in cerebellum and brain parenchyma, with multiple sites of edema on left side of parietal area suggestive of neurocysticercosis (Fig. 3).

A physician consultation was done and he was treated with intravenous injection of dexamethasone and the dose was tapered every 3 days: 4 mg dexamethasone intravenously administered thrice daily for 3 days, 2 mg intravenously administered bi-daily for 3 days, and 2 mg intravenously administered once daily for 3 days. Tablet albendazole 400 mg was prescribed bi-daily for 1 month and tablet valproic acid was prescribed 300 mg bi-daily for 1 month. Unfortunately we lost the follow-up of our patient.

## Discussion

Ocular cysticercosis is a disease with rare involvement of cutaneous skin and optic nerve. No cases have been reported in Nepal with disseminated neurocysticercosis associated with bilateral ocular involvement with papilledema. So our case should be the first case of disseminated neurocysticercosis with bilateral involvement of eye which is very rare.

Neurocysticercosis is endemic in Central and South America, Sub-Saharan Africa, and in some regions of the Far East including the Indian subcontinent, Indonesia, and China. There are very limited data on the epidemiology of cysticercosis from Nepal [3]. Soemmering reported the first case of ocular cysticercosis in 1830 [4].

Cysticercosis is the most common parasitic disease of the central nervous system in the world, but cysticercosis cutis has been reported much less frequently. Approximately 54% of the patients present with subcutaneous nodules. However, the association of neural and subcutaneous cysticercosis is not common. Less than 50 cases of disseminated cysticercosis have been reported worldwide [5]. Our patient had multiple disseminated subcutaneous nodules over his face, mandibular area, arm, elbow, and abdomen with multiple calcified cysts in a CT scan of his brain, which was suggestive of neurocysticercosis but no neurological deficit or seizure were present although symptoms of headache and dizziness were present.

A high index of suspicion is required for the diagnosis of ocular cysticercosis because of the endemic nature of this infestation in this geographic location [6]. Our case had a history of eating wild pork which is highly suspicious for neurocysticercosis.

Ocular cysticercosis may affect almost all eye tissues. The vitreous cavity, subretinal space, and subconjunctival space are common sites while involvement of other regions (such as extraocular muscle and optic nerve) is relatively less common. Although either eye may be affected, bilateral involvement is rare [2]. However, in our case there was involvement of muscles as subcutaneous nodules as well as bilateral involvement of optic nerve as bilateral papilledema which is very rare.

Similarly, another study by Vaidya *et al.* also pointed out that the clinical features of disseminated cysticercosis depend on the localization of the cysts in the organs, parasitic burden, and host parasitic interaction. The central nervous system is the most commonly involved location in disseminated cysticercosis followed by striated muscles, subcutaneous tissues, and orbits [7].

Poudel *et al.* reported probably the first case of disseminated cysticercosis in Nepal. The patient had disseminated subcutaneous nodules on bilateral temporal right medial lower arm and right chest (axillary regions). Magnetic resonance imaging revealed multiple diffuse (parenchymal, intraventricular, calcified) cysts with starry sky appearance [8]. Similarly, in our study, our patient had disseminated subcutaneous nodules on his face, mandibular area, arm, elbow, and abdomen with multiple calcified cysts in a CT scan of his brain suggestive of neurocysticercosis. However, there was an absence of papilledema unlike our study. Sune *et al.* reported a case of bipolar disorder and bilateral papilledema with diffuse parenchymatous cysticercosis demonstrated on CT scan as in our study [9]. However, in contrast, our patient had no neurological or psychological symptoms.

The diagnosis of neurocysticercosis in our study was confirmed by the presence of: one absolute criterion, that is, calcified cysts with scolex on neuroimaging; one epidemiologic criterion, that is, ingestion of pork; and one minor criterion, that is, clinical manifestations. These three criteria fulfill the diagnostic criteria of neurocysticercosis as proposed by Del Brutto *et al*. [10].

A single therapeutic approach is not expected to be useful in every patient with neurocysticercosis. Therapy usually includes a combination of symptomatic and cysticidal drugs. Surgery also has a role in the management of some patients. Albendazole has been superior to praziquantel in trials comparing the efficacy of these drugs. Cysticidal drugs must be used with caution in patients with giant subarachnoid cysticerci because the inflammatory reaction developed by the host in response to the acute destruction of the parasite may occlude leptomeningeal vessels surrounding the cyst; concomitant steroid administration is mandatory to avoid the hazard of a cerebral infarct [11]. Antiepileptic therapy may also be appropriate for patients who do not present with seizures but who are at high risk for seizures [12]. Our patient also received a combination of steroids, cysticidal drugs, and antiepileptic drugs as mentioned in the literature.

## Conclusion

Cysticercosis is an endemic disease with a diversity of clinical presentations, disseminated lesions, and unusual ocular presentations. Neurocysticercosis may be diagnosed as a cause of bilateral papilledema in endemic zones of Nepal. This case report highlights the importance of ruling out cysticercosis in cases with multiple disseminated subcutaneous nodules and bilateral papilledema.

## Data Availability

Not applicable.
